# Genetic Variations in *Angiotensinogen* Gene and Risk of Preeclampsia: A Pilot Study

**DOI:** 10.3390/jcm12041509

**Published:** 2023-02-14

**Authors:** Dong He, Xianglan Peng, Hongkai Xie, Rui Peng, Qixuan Li, Yitong Guo, Wei Wang, Hong He, Yang Chen

**Affiliations:** 1 Guangdong Provincial Key Laboratory of Pathogenesis of Heart and Spleen and Prescription Drugs Research, Department of Pharmacology, School of Chinese Pharmaceutical Science, Guangzhou University of Chinese Medicine, Guangzhou 510006, China; 2 Department of Obstetrics and Gynecology, Department of Gynecologic Oncology Research Office, The Third Affiliated Hospital of Guangzhou Medical University, Guangzhou 510150, China

**Keywords:** AGT, polymorphism, rs7079, PE susceptibility

## Abstract

Preeclampsia (PE) is a typical hypertensive disorders of pregnancy (HDP) which can cause substantial morbidity and mortality in both pregnant women and fetuses. The renin-angiotensin system (RAS) genes are the main HDP-causing genes, and Angiotensinogen (AGT) as the initial substrate can directly reflect the activity of the entire RAS. However, the association between *AGT* SNPs and PE risk has rarely been confirmed. This study was carried out to determine whether *AGT* SNPs could affect the risk of PE in 228 cases and 358 controls. The genotyping result revealed that the *AGT* rs7079 TT carrier was related to increased PE risk. Further stratified analysis illustrated that the rs7079 TT genotype significantly increased the PE risk in subgroups of Age < 35, BMI < 25, Albumin (ALB) ≥ 30 and Aspartate aminotransferase (AST) < 30. These findings demonstrated that the rs7079 might be a promising candidate SNP strongly associated with PE susceptibility.

## 1. Introduction

Hypertensive disorders of pregnancy (HDP) have long been major causes of morbidity and mortality in pregnant women, and the incidence of HDP keeps rising worldwide [1,2]. These pregnancy disorders include gestational hypertension, preeclampsia (PE), and eclampsia, which are characterized by elevated blood pressure and multiple organ disturbances, ranging from mild to severe [3,4]. The PE complicates 3–5% of all pregnancies and is estimated to result in a large number of maternal and fetal deaths globally every year [5,6]. Although the majority of PE usually resolve after delivery or in the early postpartum period, there is increasing evidence that PE confers a noteworthy increase in risks for future long-term health [7,8]. It is irrefutable that a history of PE increases the risk of hypertension, peripheral arterial disease, coronary artery disease and cerebrovascular disease in the future [9,10]. Furthermore, the severity of PE has been shown to accelerate the progression of cardiovascular disease [11]. To date, there is no stable and reliable treatment for PE. Therefore, elucidating the pathogenesis of PE is particularly critical for the diagnosis and treatment of this disease.

With the rapid development of molecular biology technology, the genetic basis of HDP has been initially revealed [12,13]. Previous studies have shown that *renalase* (*RNLS*) gene polymorphisms were associated with many diseases, such as essential hypertension, PE [14] and gestational diabetes [15]. A recent study displayed that the *interleukin 1 receptor type 1* (*IL1R1*) rs2071374G variant could lead to an increased risk of PE [16]. The *egl-9 family hypoxia inducible factor 1* (*EGLN1*) rs479200 may have the potential to become a marker to evaluate the genetic predisposition to PE [17]. However, the identified genetic mutation or SNPs can only explain the etiology of PE in a small sample of cases. Therefore, the identification and characterization of more functional mutation sites are critical to fully reveal the pathologic mechanism of PE.

The renin-angiotensin system (RAS) plays a crucial role in maintaining the homeostasis of cardiovascular function, especially the regulation of arterial blood pressure [18,19]. Its dysfunction can lead to a series of cardiovascular diseases [20,21]. Angiotensinogen (AGT), as the initial substrate in the RAS, directly reflects the activity of the entire RAS [22]. So far, the investigation that directly evaluated the effect of *AGT* SNP on PE is relatively limited. Therefore, the current study aimed to assess the association between *AGT* gene SNPs and PE risk among Chinese pregnant women.

## 2. Materials and Methods

All patients and controls in this case-control study were from the Department of Obstetrics and Gynecology, The Third Affiliated Hospital of Guangzhou Medical University, during the period from January 2016 to December 2021. 228 PE patients constituted the case group, and 358 normotensive pregnant women constituted the control group. All PE patients were diagnosed according to the diagnostic criteria of the American College of Obstetrics and Gynecology published in 2013 (systolic and/or diastolic blood pressure ≥ 140/90 mmHg after 20th week of pregnancy, plus proteinuria ≥ 300 mg per 24-h urine collection or ≥ 1+ urine dipstick; in the absence of proteinuria, hypertension in pregnancy with any of the following features: pulmnary edema, platelets count ≤ 10 × 10^10^/L, impaired liver function, systolic and/or diastolic blood pressure ≥ 160/110 mmHg, renal failure and visual disturbances) [23]. The samples of the control group and the case group were non-probability continuous and random. Normotensive pregnant women without a history of chronic hypertension and complication during pregnancy were defined as the control population. Clinical characteristics and biochemical indicators of all participants were collected for stratified analysis.

The three SNPs to be verified were selected according to the following criteria: The selected SNPs were preferentially located in 3′ and 5′ untranslated regions and exons of *AGT* gene to maximize the probability that these SNPs were functional; The minor allele frequencies of these selected SNPs should be 5% or greater. Furthermore, any two SNPs should have low linkage disequilibrium (R^2^ < 0.8). DNA of each subject for genotyping was extracted from a peripheral venous blood sample (200 µL) according to the protocol of the TIANamp Genomic DNA Kit (Tiangen Biotech, Beijing, China). The Genotyping, performed according to the instructions (Applied Biosystems, Waltham, MA, USA), was carried out in a total volume of 10 µL containing the DNA template (1 µL, 2 ng/µL), TaqMan^®^ SNP Genotyping Assay (0.06 µL, 40X), TaqPath ProAm Master Mix (3 µL) and DEPC H_2_O (add to 10 µL). The Genotyping conditions were as follows: 60 °C for 30 s, 95 °C for 5 min, followed by 40 cycles of 95 °C for 15 s and 60 °C for 1 min. The Genotyping was operated with optical 96 or 384-well plate in an ABI PRISM 7500 PCR instrument (Applied Biosystems). In addition, 5% of the samples were randomly re-tested to make sure that these re-tested samples attain 100% recurrence rate.

The student *t*-test for continuous variables was employed to evaluate clinical variables differences in the case and control groups. The χ^2^ test served to know whether these three *AGT* SNPs in the controls were in Hardy-Weinberg equilibrium (HWE). Crude ratios with respective 95% confidence intervals (CIs) and logistic regression analysis were used to assess the association between HDP risk and *AGT* SNPs. SPSS software was used to analyze all data in this plot study. The *p*-value less than 0.05 indicated that the statistical result was defined as significant.

## 3. Results

### 3.1. Clinical Characteristics of Cases and Controls

The clinical and demographic characteristics of all participants were shown in Table 1. The statistical results indicated that the body mass index (BMI), maternal age, systolic blood pressure (SBP), diastolic blood pressure (DBP), Aspartate aminotransferase (AST), Alanine aminotransferase (ALT), creatinine (CREA) and uricacid (UA) in the case group were significantly higher than that of the control group (all *p* < 0.01). On the other hand, gestational age, fetal birth weight, Albumin (ALB) and platelet count (PLT) level were significantly lower in the preeclamptic patients compared to the control subjects (all *p* < 0.01).

### 3.2. Effect of AGT Gene SNPs on PE

The genotype distributions for three selected *AGT* SNPs showed that there was a significant association between rs7079 TT genotype and PE risk (OR = 3.804, 95% CI = 1.100–13.156, *p* = 0.035) (Table 2). These results indicated that the rs7079 TT carrier shared a significantly increased risk of PE. Moreover, the *AGT* rs7079 TT genotype was associated with increased preeclampsia risk in the recessive model (OR = 4.054, 95% CI = 1.178–13.945, *p* = 0.026) (Table 2). However, we did not detect significant differences in SNP rs4762 and rs5050 in any analysis model. At last, the genotype distribution frequencies of three *AGT* SNPs of the investigated samples were in accordance with the Hardy-Weinberg equilibrium (HWE) in the control group (Table 3).

### 3.3. Stratification Analysis

In order to further clarify the role of the three SNPs in different clinical subgroups, a stratified analysis based on age, BMI and clinical parameters was performed. As presented in Table 4, the rs7079 TT genotype carriers shared significantly increased PE risk in subgroups of Age < 35 (OR = 10.988, 95% CI = 2.342–51.555, *p* = 0.001), BMI < 25 (OR = 5.153, 95% CI = 1.314–20.212, *p* = 0.024), ALB ≥ 30 (OR = 5.029, 95% CI = 1.392–18.167, *p* = 0.019) and AST < 30 (OR = 5.088, 95% CI = 1.573–16.456, *p* = 0.007).

### 3.4. The Relevance of rs7079 G>T to AGT Expression

**Figure 1:**
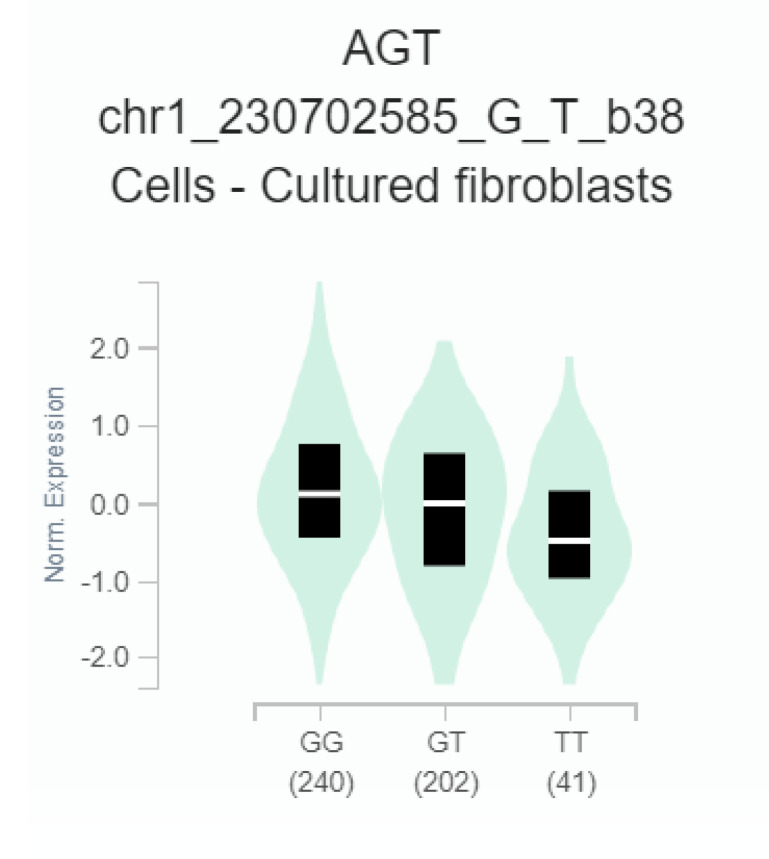
The effect of rs7079 G>T on *Angiotensinogen* (*AGT*) gene expression in cultured fibroblasts (*p* = 0.000099). The gene expression data was obtained from the public database GTEx portal.

To determine whether rs7079 could lead to change of *AGT* gene expression, the GTEx database was used to verify the correlation between rs7079 G>T and *AGT* mRNA expression. The results showed that the rs7079 T allele was significantly related to the decrease of *AGT* expression level in cultured fibroblasts (*p* = 0.000099) (Figure 1).

## 4. Discussion

To date, the *AGT* gene has been confirmed to be closely related to the occurrence and development of various types of diseases, including cardiovascular disease [24] and colorectal cancer [25]. A recent experimental study pointed out that loss of AGT protein in specific cells can improve high-fat diet-induced insulin tolerance [26]. And Yilmaz et al. reported that urinary AGT levels in 35-week gestational women with PE were significantly elevated compared to normal pregnancies and non-pregnant women [27]. Moreover, consistent evidence found by clinical research showed that plasma-derived oxidized AGT in pregnant women with PE retained a dominant level compared to normotensive controls [28]. These above results indicated the important biological function of AGT in the process of diseases occurrence and development.

Contributions of *AGT* SNPs to cardiovascular diseases have been widely accepted in recent years [29,30]. In patients with peripheral arterial disease, the association between *AGT* rs699 CC genotype and high-density lipoprotein (HDL) levels was proved to be significant [31]. The non-alcoholic fatty liver disease (NAFLD) patients with the *AGT* rs5051 TC + CC genotype had a significantly increased risk of coronary heart disease (CHD) in the northern Chinese Han population [32]. Nonetheless, the association between these three *AGT* SNPs and PE has not been well established. Through genotyping and stratified analysis of 228 cases and 358 controls, the present study successfully clarified the association between *AGT* SNPs and PE risk. The rs4762 accompanied by the mutation from threonine to methionine at residence 174 is a typical missense mutation of *AGT* gene [33]. Previous study confirmed that the rs4762 showed a significant risk for the diabetes mellitus in transplant patients [34]. Another clinical study in Egyptians pointed out the rs4762 variant may increased the risk for end-stage renal failure risk [35]. Compared with rs4762, the *AGT* rs5050 leads to the transformation from adenine to cytosine in the promoter region of gene. The *AGT* rs5050 GG carriers with astrocytoma were more likely to have poor prognosis [36]. In children with Kawasaki Disease, rs5050 T>G was associated with the risk of coronary artery aneurysm [37]. However, our findings indicated that there was no statistically significant association between these two SNPs (rs4762 and rs050) and PE susceptibility (Table 2). We speculated that the two functional SNP sites rs4762 and rs050 would not affect the dynamic balance of RAS, so that the susceptibility of PE did not change. The *AGT* rs7079 located in the 3′UTR is the direct binding site of miR-31 and miR-584 regulating *AGT* gene expression [38,39]. Moreover, the genotyping results in this study showed that the rs7079 TT genotype was related to increased PE risk (Table 2). The stratified analysis further indicated that the rs7079 TT genotype carriers shared significantly increased PE risk in subgroups of Age < 35, BMI < 25, ALB ≥ 30 and AST < 30 (Table 4). Moreover, we found that the rs7079 T allele resulted in the decrease of *AGT* expression level in the GTEx portal (Figure 1). Based on the above findings, we speculated that the rs7079 T allele may increase PE risk by disturbing the dynamic equilibrium of the RAS.

There are some deficiencies as follows that could be improved in future research. First, this study only verified the function of three *AGT* SNPs. More *AGT* SNPs should be tested to comprehensively evaluate the association between *AGT* SNPs and PE risk. Second, in consideration of this study’s relatively limited sample size, larger sample sizes are needed to confirm the impact of *AGT* SNPs on PE risk. Third, the non-genetic risk factors, including environmental factors, life style, and health care, are also considered as the main nosogenesis of PE [40,41]. Therefore, the non-genetic risk factors merit further research in combination with the analysis between the PE risk and SNPs. Finally, the genetic diversity among different ethnic groups also affects the susceptibility to PE. In the subsequent evaluation of the association between *AGT* gene polymorphisms and susceptibility to PE, it will be a great challenge to exclude functional genetic diversity that may potentially affect the risk of PE.

## Figures and Tables

**Table 1:** Clinical characteristics overview of the study population.

Characteristics	Control (*n* = 358)	Case (*n* = 228)	*p*
BMI (kg/m^2^)	20.95 ± 3.07	23.68 ± 4.33	**0.000**
Maternal age (years)	28.94 ± 4.58	32.89 ± 5.50	**0.000**
SBP (mm Hg)	117.16 ± 6.44	142.07 ± 11.11	**0.000**
DBP (mm Hg)	72.25 ± 4.75	89.93 ± 8.79	**0.000**
Gestational age (weeks)	39.10 ± 1.14	34.36 ± 4.27	**0.000**
Fetal birth weight (g)	3218.62 ± 404.27	2171.32 ± 980.26	**0.000**
ALB (g/L)	35.46 ± 2.76	29.38 ± 4.64	**0.000**
AST (U/L)	15.305 ± 4.31	29.22 ± 59.76	**0.001**
PLT (×10^9^/L)	232.39 ± 60.24	209.11 ± 73.00	**0.000**
ALT (U/L)	9.45 ± 4.89	23.379 ± 60.85	**0.000**
CREA (mg/dl)	51.30 ± 9.45	66.93 ± 23.81	**0.000**
UA (μmol/L)	333.35 ± 83.94	450.81 ± 136.00	**0.000**

BMI, body mass index; SBP, systolic blood pressure; DBP, diastolic blood pressure; ALB, Albumin; AST, aspartate amino transferase; PLT, platelet count; ALT, Alanine aminotransferase; CREA, creatinine; UA, uricacid. Data were presented as mean ± SD. Significant findings (*p* values less than 0.05) were shown in bold font.

**Table 2:** Genotype and allelic distribution between PE and healthy controls.

Genetype	Control (*n* = 358)	Case (*n* = 228)	Crude OR (95% CI)	*p*	Adjusted OR (95% CI)	*p* ^a^
rs4762 G > A						
GG	284	182		1.000		1.000
AG	68	40	0.918 (0.596–1.415)	0.698	0.917 (0.568–1.480)	0.723
AA	6	6	1.560 (0.496–4.912)	0.447	1.318 (0.373–4.658)	0.668
Dominant	74	46	0.970 (0.642–1.465)	0.885	0.953 (0.604–1.505)	0.837
Recessive	352	222	1.586 (0.505–4.978)	0.430	1.340 (0.380–4.720)	0.649
rs5050 T > G						
TT	250	163		1.000		1.000
GT	98	58	0.908 (0.621–1.327)	0.617	0.829 (0.543–1.266)	0.385
GG	10	7	1.074 (0.401–2.877)	0.888	1.009 (0.343–2.962)	0.987
Dominant	108	65	0.923 (0.640–1.330)	0.668	0.846 (0.563–1.271)	0.420
Recessive	348	221	1.102 (0.413–2.938)	0.846	1.062 (0.364–3.099)	0.912
rs7079 G > T						
GG	271	172		1.000		1.000
GT	83	42	0.797 (0.525–1.210)	0.287	0.753 (0.472–1.201)	0.233
TT	4	14	5.515 (1.786–17.029)	**0.003**	3.804 (1.100–13.156)	**0.035**
Dominant	87	56	1.014 (0.689–1.492)	0.943	1.095 (0.708–1.694)	0.684
Recessive	354	214	5.790 (1.881–17.817)	**0.002**	4.054 (1.178–13.945)	**0.026**

OR, odds ratio; CI, confidence interval; *p* ^a^, adjusted for age and BMI. Logistic regression analysis and the chi-square test were performed for genotype and allelic distribution between PE and healthy controls. Significant findings (*p* values less than 0.05) were shown in bold font.

**Table 3:** Hardy–Weinberg equilibrium of three *AGT* SNPs genotype distribution frequency in the control group.

	Expected			Observed			χ^2^	*p*
rs4762 G > A	GG	AG	AA	GG	AG	AA	0.664	0.717
Control	282.47	71.06	4.47	284	68	6		
rs5050 T > G	TT	GT	GG	TT	GT	GG	0.011	0.994
Control	249.72	98.55	9.72	250	98	10		
rs7079 G > T	GG	GT	TT	GG	GT	TT	0.721	0.697
Control	272.78	79.43	5.78	271	83	4		

Comparisons were performed with the chi-square test.

**Table 4:** Stratified analysis for PE susceptibility and *AGT* rs7079 G > T.

Variables	rs7079			*p*
	(Cases/Controls)		OR (95%CI)	
	TT	GG/GT		
Age				
<35	9/2	129/315	10.988(2.342–51.555)	**0.001**
≥35	5/2	85/39	1.147(0.213–6.174)	1.000
BMI				
<25	7/3	144/318	5.153 (1.314–20.212)	**0.024**
≥25	7/1	70/36	3.600 (0.426–30.400)	0.391
ALB				
<30	8/0	112/12	/	1.000
≥30	6/4	102/342	5.029 (1.392–18.167)	**0.019**
AST				
<30	10/4	171/348	5.088(1.573–16.456)	**0.007**
≥30	4/0	43/6	/	1.000

BMI, body mass index; ALB, Albumin; AST, aspartate amino transferase. OR, odds ratio; CI, confidence interval. Chi-squared test were performed for Stratified analysis. Significant findings (*p* values less than 0.05) were shown in bold font.

## Data Availability

The data presented in this study are available on request from the corresponding author.
